# Understanding the Role of the Lateral Dimensional Property of Graphene Oxide on Its Interactions with Renal Cells

**DOI:** 10.3390/molecules27227956

**Published:** 2022-11-17

**Authors:** Wei Chen, Bing Wang, Shanshan Liang, Meng Wang, Lingna Zheng, Si Xu, Jiali Wang, Hao Fang, Pu Yang, Weiyue Feng

**Affiliations:** 1 CAS Key Laboratory for Biomedical Effects of Nanomaterials and Nanosafety, Institute of High Energy Physics, Chinese Academy of Sciences, Beijing 100049, China; 2 University of Chinese Academy of Sciences, Beijing 100049, China; 3 School of Pharmacy, Yantai University, Yantai 264005, China

**Keywords:** graphene oxide, lateral dimension, renal cells, interaction, cytotoxicity

## Abstract

Renal excretion is expected to be the major route for the elimination of biomedically applied nanoparticles from the body. Hence, understanding the nanomedicine–kidney interaction is crucially required, but it is still far from being understood. Herein, we explored the lateral dimension- (~70 nm and ~300 nm), dose- (1, 5, and 15 mg/kg in vivo and 0.1~250 μg/mL in vitro), and time-dependent (48 h and 7 d in vivo) deposition and injury of PEGylated graphene oxide sheets (GOs) in the kidney after i.v. injection in mice. We specially investigated the cytotoxic effects on three typical kidney cell types with which GO renal excretion is related: human renal glomerular endothelial cells (HRGECs) and human podocytes, and human proximal tubular epithelial cells (HK-2). By using in vivo fluorescence imaging and in situ Raman imaging and spectroscopic analysis, we revealed that GOs could gradually be eliminated from the kidneys, where the glomeruli and renal tubules are their target deposition sites, but only the high dose of GO injection induced obvious renal histological and ultrastructural changes. We showed that the high-dose GO-induced cytotoxicity included a cell viability decrease and cellular apoptosis increase. GO uptake by renal cells triggered cellular membrane damage (intracellular LDH release) and increased levels of oxidative stress (ROS level elevation and a decrease in the balance of the GSH/GSSG ratio) accompanied by a mitochondrial membrane potential decrease and up-regulation of the expression of pro-inflammatory cytokines TNF-α and IL-18, resulting in cellular apoptosis. GO treatments activated Keap1/Nrf2 signaling; however, the antioxidant function of Nrf2 could be inhibited by apoptotic engagement. GO-induced cytotoxicity was demonstrated to be associated with oxidative stress and an inflammation reaction. Generally, the *l*-GOs presented more pronounced cytotoxicity and more severe cellular injury than *s*-GOs did, demonstrating lateral size-dependent toxicity to the renal cells. More importantly, GO-induced cytotoxicity was independent of renal cell type. The results suggest that the dosage of GOs in biomedical applications should be considered and that more attention should be paid to the ability of a high dose of GO to cause renal deposition and potential nephrotoxicity.

## 1. Introduction

Graphene oxide (GO) has attracted great interest in biomedical applications due to its excellent flexibility and dispersion, biocompatibility, large specific surface area, various functional groups on the surface, and many other unique physicochemical properties [1,2,3,4,5]. Thus, GO has been shown to be promising for targeted drug delivery [6] and as an immune activator [7], biosensor [8], and so forth [1,2]. Graphene family materials can be used as high-performance carriers in the biomedical field for anticancer drug delivery due to an extremely high drug-loading capacity, which is superior to other materials. In addition, due to the advantage of the large, modified surface of graphene-based materials, the diagnostic and therapeutic agents can be concentrated on an all-in-one graphene-based platform, which makes the simultaneous development of personalized and multifunctional smart platforms for diagnosis and therapy possible [9,10,11]. The kidney is the principal excreting organ for most prescribed drugs and nanoparticles (NPs) [12,13]. It is generally believed that NPs with a diameter smaller than 6 nm can be cleared by the kidney because of their ability to readily pass through the glomerular filtration barrier [12,13,14]. As for the two-dimensional (2D) nanomaterials, such as graphene oxide nanosheets (GOs), the processes of their renal clearance might be complicated. Recently, several studies confirmed that the intravenously injected GOs could be eliminated rapidly from the kidney and excreted in urine by the rolling, crumpling, or folding of their morphology [15,16], which might be due to the rapid uptake and subsequent release of GOs by podocyte cells [15], while a small amount of GOs might be deposited in the kidney in the medium to long term [16,17]. So far, the impact of GOs on the kidney is not yet fully understood. In particular, the excretion of nanomaterials from the kidney is an integrated process involving glomerular filtration, tubular secretion, and reabsorption [12]. Therefore, it is an urgent need to explore the interactions of GOs with various renal cells and the role of the lateral size of GOs in the interactions.

To assess the nephrotoxicity of nanomaterials, the biological effects of nanomaterials on diverse renal cell types need to be understood first. The kidneys are rich in blood flow, with blood flowing from the kidneys through the main renal artery to the glomerulus, then traveling through the capillaries surrounding the renal tubules, and finally converging again to the main renal vein [18]. Thus, nanoparticles passing through the kidney may participate in all these renal transport pathways. Three major renal cells, including glomerular vascular endothelial cells, podocytes, and tubular epithelial cells, play key roles in the glomerular filtration, tubular secretion, and tubular reabsorption processes. The interactions of some metal-based NPs with renal cells have been reported [12,19]. Interestingly, Weng et al. [19] reported that ceria nanoparticles (CNPs) could prevent chemotherapy-induced acute kidney injury (AKI) because CNP catalytically decomposed hydrogen peroxide and activated the Nrf2/Keap1 signaling pathway, which restored the redox homeostasis of renal tubules. Several studies reported that GOs could induce membrane damage [20], lipid peroxidation [21], and inflammatory responses [22] when GOs entered biological systems. The performance of GOs in biological environments has a strong association with their physiochemical properties. Gholami et al. found that functionalization of the surface of GO nanosheets with chitosan and a carboxylate group significantly reduced its toxicity to dental pulp stem cells [23]. Functionalized GOs with chitosan were also found to protect against enzymatic cleavage and retain collagenase activity [24]. Li and his colleagues [25] demonstrated that the lateral size of GOs determined differential cellular uptake and GSDMD-mediated pyroptosis in liver cells. Yue et al. [26] also reported that the micro-sized GOs presented divergent intracellular locations and induced much stronger inflammation responses than the nanosized GOs. In addition, the surface oxidation state and carbon radical content of GOs were demonstrated to play major roles in the induction of toxicity in mammalian cells [21]. However, the mechanical interactions of GOs with renal cells and the contribution of the GO physicochemical properties are still seldom studied.

Herein, we prepared two lateral-sized PEGylated GOs (*s*-GOs and *l*-GOs) for the exploration of lateral dimension-, dose- and time-dependent deposition and injury in mouse kidneys after GO intravenous injection. Further, three human-derived renal cells, including human renal glomerular endothelial cells (HRGECs), human podocytes, and human proximal tubule epithelial cells (HK-2), which represent the major target cells during GO renal clearance, were selected for investigating the role of the lateral dimension of GOs in the cytotoxicity. We demonstrated that the lateral size of GOs played a key role in the high-dose GO-induced nephrotoxicity and contributed to the oxidative stress-related toxic mechanism.

## 2. Results

### 2.1. Physiochemical Properties of Graphene Oxide Nanosheets (GOs)

**Figure 1:**
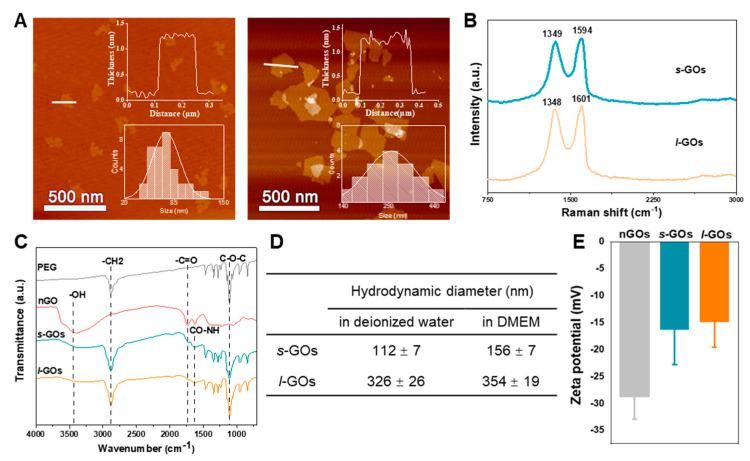
Physicochemical characterization of *s*-GOs and *l*-GOs. (**A**) AFM images of *s*-GOs and *l*-GOs. The insert figures show the distribution of thickness and lateral dimensions of GOs. (**B**) Raman spectra of *s*-GOs and *l*-GOs. (**C**) FTIR spectra of PEG, nGOs, *s*-GOs, and *l*-GOs. (**D**) Hydrodynamic diameter of *s*-GOs and *l*-GOs measured by DLS in water and in DMEM. (**E**) Zeta potentials of *s*-GOs and *l*-GOs in deionized water measured by DLS. nGOs: non−PEGylated GOs.

The atomic force microscope (AFM) analysis showed that the thickness of both the thin PEGylated *s*-GOs and *l*-GOs was approximately 1 nm, demonstrating a single layer of the GOs. The average lateral dimensions of *s*-GOs and *l*-GOs were ~70 nm and ~300 nm, respectively (Figure 1A). Both lateral sizes are in a comparable size range (usually from ~50 to ~1000 nm) for GOs in biomedical applications. The typical Raman spectra of GO were characterized by a D band at ca. 1348~1349 cm^−1^ and a G band at ca. 1594~1601 cm^−1^ (Figure 1B). The FT-IR spectrum of GO showed strong bands at ~3431 cm^−1^ (-OH), ~1739 cm^−1^ (O=C), and ~1106 cm^−1^ (C-O-C), indicating the presence of oxygen-containing groups, such as hydroxyl, carboxylic, and epoxy groups, in GO. The decreased intensity of the carboxylic peak and the appearance of the -CH_2_ band (2881 cm^−1^) and the amide-carbonyl (-CO-NH-) stretching vibration (~1635 cm^−1^) in PEGylated *s*-GOs and *l*-GOs suggested that NH_2_-PEG-NH_2_ was successfully covalently conjugated with *s*-GOs and *l*-GOs (Figure 1C). The hydrodynamic diameter of *s*-GOs was 112 nm in deionized water and 156 nm in DMEM culture medium; and for *l*-GOs, the diameters were 326 nm and 354 nm, respectively (Figure 1D). The zeta potentials of non-PEGylated GOs (nGOs), *s*-GOs, and *l*-GOs in deionized water were 28.6 mV, −16.2 mV, and −14.6 mV, respectively, indicating the negatively charged surface of the GOs (Figure 1E) and the successful attachment of PEG to the graphene oxide surface [27]. In addition, the PEGylated-GOs showed better dispersion stability in 0.9% NaCl solution and DMEM culture medium, while nGOs underwent significant aggregation at 4 h (Figure S1).

### 2.2. Renal Clearance of GOs after i.v. Injection

**Figure 2:**
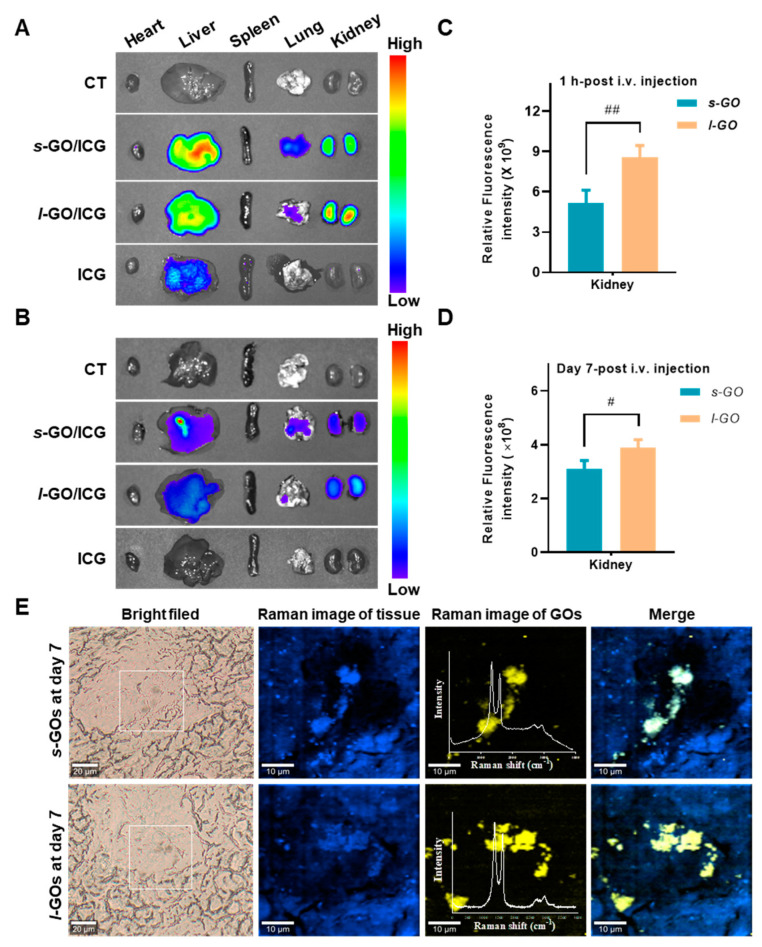
Representative ex vivo fluorescence images of heart, liver, spleen, lung, and kidneys at 1 h (**A**) and day 7 (**B**) after i.v. injection of *s*-GO/ICG and *l*-GO/ICG at 5 mg/kg bw dosage. Quantitative analysis of fluorescence intensity of GO/ICG in kidney at 1 h (**C**) and day 7 (**D**) after i.v. injection. The unit of average radiant efficiency ([p/s/cm^2^/sr]/[µw/cm^2^]) is the ratio of the radiant flux of the emitted radiation to the power consumed by the source. # *p* < 0.05, ## *p* < 0.01 for *s*-GO− vs. *l*-GO− treated group. (**E**) Confocal Raman images show GOs deposited in the renal cortex after 7 days of the i.v. injection of 15 mg/kg bw *s*-GOs and *l*-GOs. From left to right: Bright field (BF) image; Raman image (RI) of kidney tissue (blue color); Raman spectrum analysis shows the characteristic D and G bands of GOs (yellow color) in the renal cortex; merge image of tissue and GOs.

GO/ICG was used as a fluorescent probe to track the biodistribution of GOs in mice after i.v. injection. The ex vivo imaging results clearly demonstrated the aggregation of *s*-GO and *l*-GO in the kidney at 1 h and day 7 post-injection **(**Figure 2A,B). The semi-quantitative analysis showed that from 1 h to day 7 post-injection, the quantity of GOs in the kidneys obviously decreased, indicating the renal elimination of GOs. Comparatively, the quantity of *l*-GOs deposited in the kidneys was significantly higher than that of *s*-GOs **(**Figure 2C,D).

The deposition of GOs in the mouse kidneys was further identified using in situ Raman imaging and spectroscopic analysis. In the renal cortex, Raman spectroscopy showed the characteristic D band at 1345 cm^−1^ and G bands at 1595 or 1597 cm^−1^ of GOs after 7 days of the injection (Figure 2E), suggesting GOs were partially deposited in the kidney.

### 2.3. Pathological Changes in the Glomeruli and Renal Tubules

**Figure 3:**
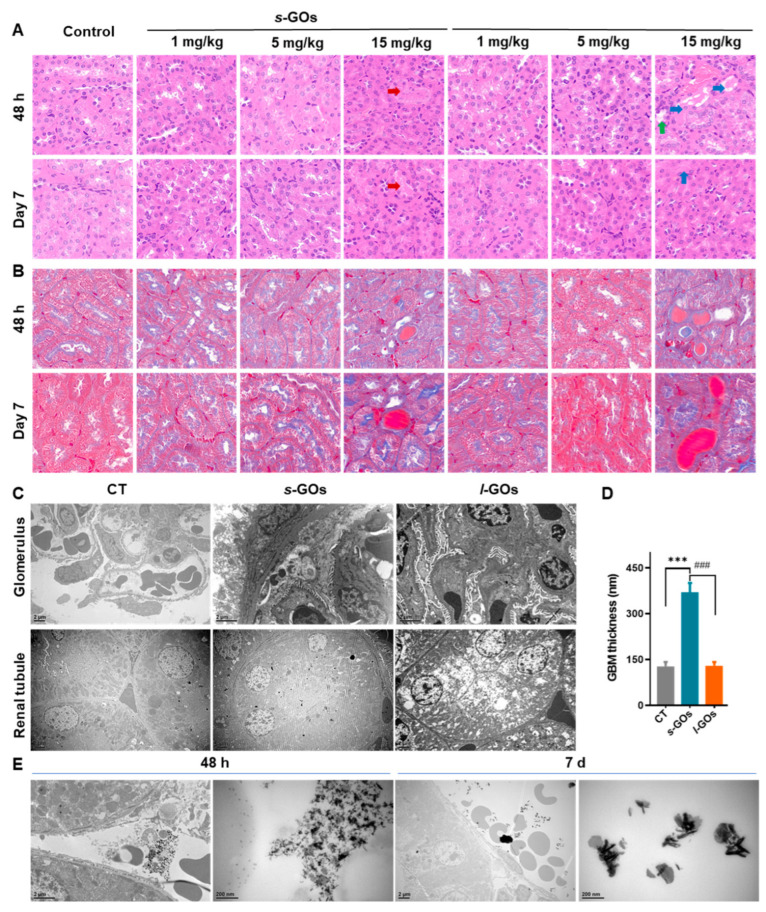
(**A**) Representative light microscopy images of H&E-stained kidney sections harvested from *s*-GO− and *l*-GO−treated mice at 48 h and day 7 after i.v. injection. There was a moderate dilatation of the tubules containing hyaline (protein) casts in the renal tubules at 48 h and day 7 after 15 mg/kg *s*-GO and *l*-GO administration. No obvious pathological changes were found in the kidneys of 1 and 5 mg/kg GO− treated mice. (**B**) Masson’s trichrome staining of kidney tissues of *s*-GO− and *l*-GO−treated mice at 48 h and day 7 after i.v. injection. No evidence of fibrosis was observed. (**C**) TEM micrographs from glomerulus and renal tubule at day 7 after mice i.v. injection of 15 mg/kg bw *s*-GOs and *l*-GOs. (**D**) Statistical results of glomerular basement membrane thickness. Red arrow: necrosis of renal tubular epithelial cells. (**E**) TEM images show *l*-GOs deposited in the renal interstitial capillaries after i.v. injection of 15 mg/kg bw at 48 h and 7 days. Green arrow: dilatation of the tubules. Blue arrow: cast formation. *** *p* < 0.001 for GO−treated vs. the control group; ### *p* < 0.001 for *s*-GO− vs. *l*-GO−treated group.

The histological examinations of the mouse kidneys were performed after 48 h and 7 days of the i.v. injection of GOs (Figure 3). The H&E-stained images showed that no obvious pathological changes were induced in the kidneys after treatments with 1 and 5 mg/kg bw *s*-GO and *l*-GO. In contrast, in the group of mice that was administered with a high dose (15 mg/kg bw) of *s*-GOs and *l*-GOs, a moderate dilatation of the tubules, hyaline (protein) casts in the tubule, and slight damage to the tubular epithelium were found (Figure 3A). Masson’s trichrome staining selectively stains the muscle, erythrocytes, and cytoplasm red, collagen blue, and nuclei dark brown in the examination of histopathological features of the kidney. Here, no evidence of fibrosis was observed in the kidney at 48 h and 7 days post-injection (Figure 3B).

Further ultrastructural observation of renal glomeruli and tubules by transmission electron microscopy (TEM) showed that the thickness of the glomerular basement membrane obviously increased on the 7th day after injection of 15 mg/kg *s*-GOs compared with the control mice (Figure 3C,D), which was about 3-fold higher than those in the control and *l*-GO groups. Moreover, vacuolated mitochondria were observed in the renal tubular epithelial cells of *l*-GO-administrated mice (Figure 3C). An obvious deposition of GOs was found in the renal interstitial capillaries of 15 mg/kg *l*-GO-treated mice (Figure 3E).

### 2.4. High-Dose GO-Treatment-Induced Cytotoxicity and Apoptosis in HRGECs, Human Podocyte Primary Cells, and HK-2 Cells

**Figure 4:**
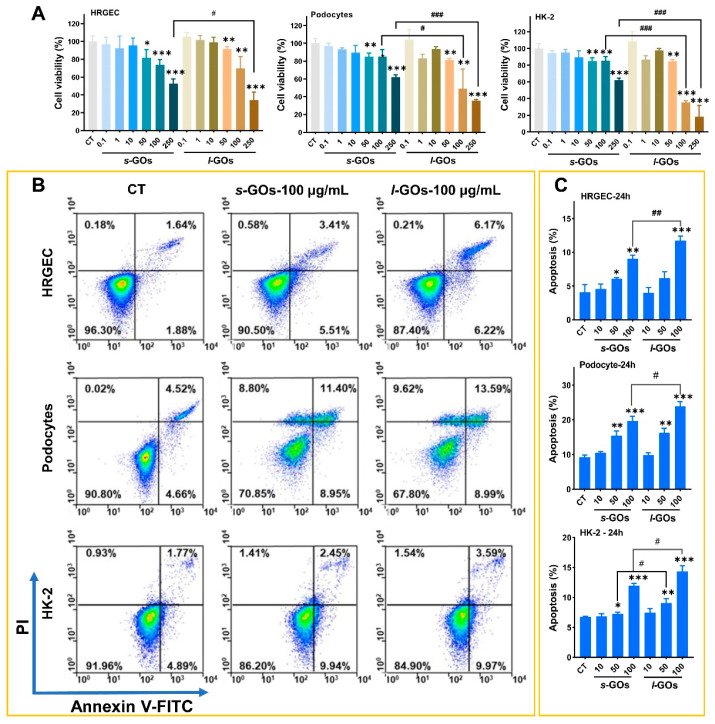
Cytotoxic assays of *s*-GO and *l*-GO treatments on HRGECs, human podocytes, and HK-2 cells. (**A**) Cell viability assays of HRGECs, podocytes, and HK-2 cells following *s*-GO and *l*-GO treatments at 0.1, 1, 10, 50, 100, and 250 μg/mL doses for 24 h. (**B**) Annexin V-FITC/PI flow cytometry for detection of apoptosis in cells treated with 100 μg/mL *s*-GOs and *l*-GOs for 24 h. (**C**) Percentage of apoptotic HRGECs, podocytes, and HK-2 cells. * *p* < 0.05, ** *p* < 0.01, and *** *p* < 0.001 for GO-treated vs. the control group; ^#^ *p* < 0.05, ^##^ *p* < 0.01, and ^###^ *p* < 0.001 for s-GO− vs. *l*-GO−treated group.

The above in vivo results showed that the GOs deposited and induced injury in the glomeruli and renal tubules. Thus, to evaluate the potential cytotoxicity to renal cells during GO accumulation and excretion in the kidney, three human renal cells, including human renal glomerular endothelial cells (HRGECs), human podocyte primary cells, and the human renal proximal tubule epithelial cell line (HK-2), were selected for investigation. The CCK-8 assay showed that with 0.1~250 μg/mL *s*-GO or *l*-GO treatments, only the relatively high dose, i.e., doses >50 μg/mL, induced obvious time- and dose-dependent decreases in cell viability in all three renal cells **(**Figure 4A and Figure S2). Comparatively, the *l*-GO treatment led to more severe cytotoxicity than the *s*-GO did because a significantly higher inhibition of cell growth was induced by *l*-GOs under the corresponding dose and time than by *s*-GOs. Furthermore, the highest decrease in cell viability was detected in the HK-2 cells after GO treatments. The 24 h 250 μg/mL *l*-GO treatments induced 65.9%, 64.4%, and 72.0% inhibition of HRGECs, podocytes, and HK-2 cell growth, respectively.

Additional apoptosis analysis showed that the 50 and 100 μg/mL doses of GO treatments initiated significant apoptosis in the three renal cells, whereas no significant apoptosis was induced by the GO treatments of a low dose of 10 μg/mL (Figure 4B and Figure S3). The 100 μg/mL *s*-GO treatments for 24 h led to 9.0%, 19.6%, and 12% apoptosis in HRGECs, podocytes, and HK-2 cells, respectively; as for *l*-GO treatments, apoptosis was detected as 11.7%, 23.9%, and 14.4% in the respective renal cells (Figure 4C). Similar to the results of the cell viability assay, the degree of apoptosis induced by *l*-GO treatments was higher than that by *s*-GOs.

### 2.5. High Dose of GO Treatments Induced Intracellular LDH Release and Oxidative Stress in HRGECs, Podocytes, and HK-2 Cells

**Figure 5:**
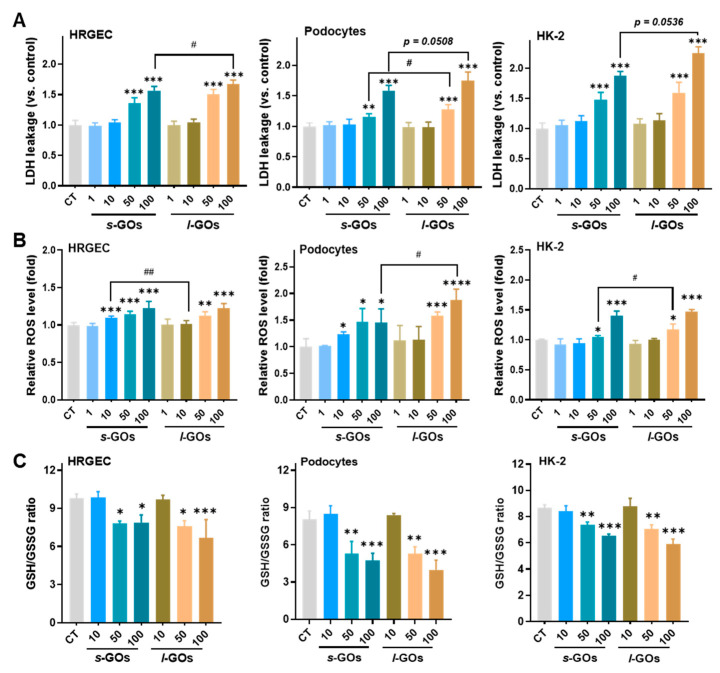
(**A**) LDH leakage in HRGECs, podocytes, and HK-2 cells was determined after cells were treated with *s*-GOs and *l*-GOs for 24 h. (**B**) The intracellular ROS levels were caused by 2 h of *s*-GO and *l*-GO treatments. (**C**) GSH/GSSG ratios in HRGECs, podocytes, and HK-2 cells after 2 h treatments with *s*-GOs and *l*-GOs. * *p* < 0.05, ** *p* < 0.01, *** *p* < 0.001, and **** *p* < 0.0001 for GO−treated group vs. the control group; ^#^ *p* < 0.05 and ^##^ *p* < 0.01 for *s*-GO− vs. *l*-GO−treated group.

GOs with sharp edges have been reported to disrupt the integrity of cell membranes and thus cause cell damage when they interact with cell membranes [20,21]. LDH release is a common marker of cell membrane damage. Here, we found that with the decrease in cell viability, the levels of LDH leakage from the renal cells significantly increased after 6 h or 24 h treatments with 100 μg/mL *s*-GOs and *l*-GOs (Figure 5A and Figure S4). It is noteworthy that a significant elevation of LDH release in HK-2 cells was observed even at 6 h of 50 μg/mL GO treatments; in addition, the levels of released LDH in HK-2 cells were higher than in the other two renal cell types, suggesting that they are more sensitive to the GO treatments. Similarly, the treatments with *l*-GOs led to more statistical LDH leakage in renal cells than those with *s*-GO.

Intracellular redox homeostasis is essential in the maintenance of cellular processes. Reactive oxygen species (ROS) generation is one of the most frequent causes of NP-related cytotoxicity due to the ultrahigh biological/chemical surface activity of NPs [21,23]. In this study, the renal cells showed sensitivity in the production of ROS to GO treatments that even with 2 h of 10 μg/mL *s*-GO treatment in HRGECs and podocyte cells, significantly increased levels of ROS were detected (Figure 5B). Meanwhile, the ratio of GSH/GSSG significantly decreased in the renal cells with 2 h of 50 and 100 μg/mL *s*-GO and *l*-GO treatments.

### 2.6. Cellular Uptake of GOs in HK-2 Cells

As it is known, for drug elimination, proximal tubular secretion undertakes the primary role for renal clearance of commonly prescribed medications [28]. Thus, investigation of the interactions of GOs with renal proximal tubular cells is important for the understanding of GO renal elimination and induced nephrotoxicity.

**Figure 6:**
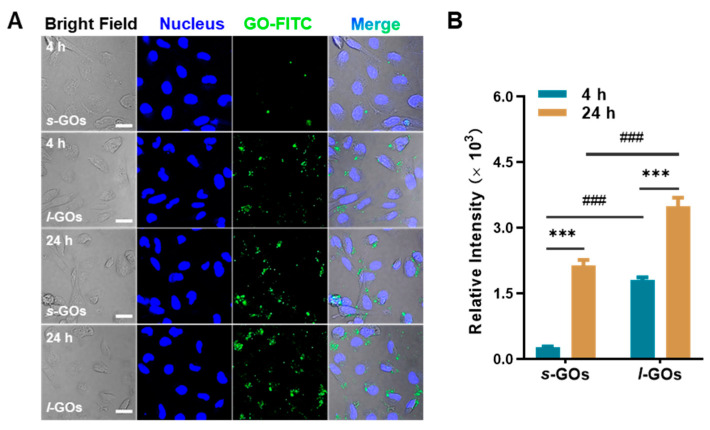
(**A**) Cellular uptake of *s*-GOs and *l*-GOs in HK-2 cells was observed by confocal laser scanning microscopy. HK-2 cells were co-cultured with 10 μg/mL *s*-GO/FITC and *l*-GO/FITC for 4 h and 24 h. Nucleus was stained by DAPI (blue); GOs were labeled by FITC-BSA (green). Scale bar = 20 μm. (**B**) Quantitative results of cellular uptake of GO/FITC in HK-2 cells. *** *p* < 0.001 indicates a significant difference between GO−treated group and the control group. ### *p* < 0.001 indicates a significant difference between *s*-GO− and *l*-GO−treated groups.

We found that after HK-2 cells were co-cultured with 10 μg/mL of GOs for 4 h or 24 h, the cellular uptake of *l*-GOs was significantly higher than that of *s*-GOs, and the intracellular amount of *s*-GOs and *l*-GOs significantly increased when the treatment time was extended to 24 h (Figure 6).

### 2.7. High Dose of GO Treatments Induced Decrease in Mitochondrial Membrane Potential, Elevation of mRNA Expression for Oxidative Stress, and Pro-Inflammatory Cytokines

**Figure 7:**
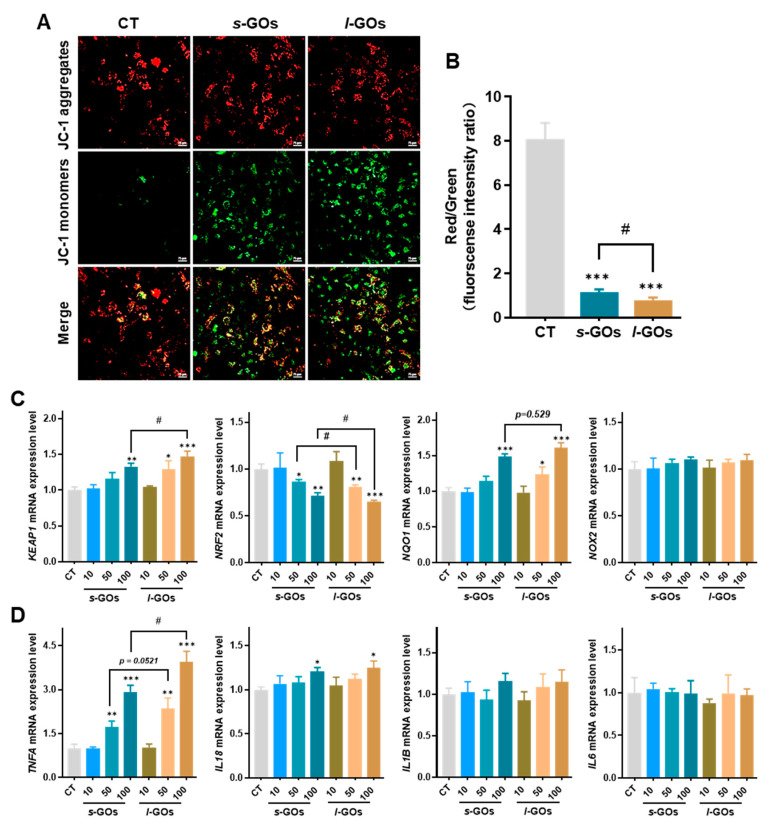
The biological effects of GO treatments on HK-2 cells. (**A**) Decreased mitochondrial membrane potential was examined in *s*-GO− and *l*-GO−treated HK-2 cells using confocal laser scanning microscopy. HK-2 cells were treated with 100 μg/mL *s*-GOs and *l*-GOs for 2 h. Scale bar = 40 μm. (**B**) Quantitative analysis of red/green fluorescence intensity ratio. (**C**) *KEAP1*, *NRF2*, *NQO1,* and *NOX2* mRNA expression in HK-2 cells after cells were co-cultured with *s*-GOs and *l*-GOs for 24 h. (**D**) Inflammatory genes *TNFA*, *IL18*, *IL1B*, and *IL6* mRNA expression in HK-2 cells after cells were co-cultured with *s*-GOs and *l*-GOs for 24 h. * *p* < 0.05, ** *p* < 0.01, and *** *p* < 0.001 for GO−treated vs. the control group; # *p* < 0.05 for *s*-GO− vs. *l*-GO−treated group.

The mitochondrial membrane potential is an essential component in the process of energy storage during oxidative phosphorylation. The loss of mitochondrial membrane potential may be associated with ROS production and oxidative stress-induced cell death. As shown in Figure 7A, normal cells with high mitochondrial membrane potential were observed as red signals (JC-1 aggregates); however, after HK-2 cells were treated with 100 μg/mL *s*-GOs and *l*-GOs for 24 h, abundant green signals (JC-1 monomers) were observed, suggesting a depolarized mitochondrial membrane due to the blockage of the mitochondrial electron transfer chain. Compared to the untreated cells, the red/green fluorescence intensity ratio was calculated to show a 7-fold and 10-fold acute reduction in *s*-GO- and *l*-GO-treated cells, respectively (Figure 7B). Comparatively, the decrease in the red/green fluorescence ratio in *l*-GO-treated HK-2 cells was significantly higher than that in *s*-GO cells.

The Kelch-like ECH-associated protein 1 (Keap1)–nuclear factor erythroid2-related factor 2 (Nrf2) signaling pathway is one of the most important cellular antioxidant systems. To investigate whether the Nrf2/Keap1 signaling pathway was activated for ROS scavenging, the mRNA expression of *KEAP1*, *NRF2*, the Nrf2 target gene NAD(P)H: quinone oxidoreductase 1 (*NQO1*), and NADPH oxidase 2 (*NOX2*) were further measured by RT-qPCR. The data showed that the mRNA expression for *KEAP1* was significantly elevated and that *NRF2* significantly decreased in HK-2 cells with 100 μg/mL *s*-GO or *l*-GO treatments, suggesting the dissociation of the Nrf2/Keap1 complex (Figure 7C). Meanwhile, the treatments led to a significant up-regulation of the mRNA level of *NQO1*, presenting in a dose–response manner. The larger changes in the mRNA levels were observed in *l*-GO-treated cells than in *s*-GO ones. However, no statistical alteration of the expression for *NOX2* was observed.

Oxidative stress can lead to chronic inflammation, which is one of the mechanisms for nanomaterial-induced cytotoxicity. To elucidate the role of inflammatory factors in GO-induced cytotoxicity, the mRNA expression for pro-inflammatory cytokines including TNF-α, IL-1β, IL-6, and IL-18 was determined (Figure 7D). The results showed a dose–dependent up-regulation of mRNA expression for *TNFA* and *IL18*, and significant changes were detected in the cells with 50 or 100 μg/mL GO treatments. No significant changes in the expression levels of *IL*-1β and *IL6* were detected.

## 3. Discussion

Graphene oxide has been used as a multifunctional pharmaceutical delivery platform for biomedical applications such as anticancer drug and gene delivery, biosensors, bioimaging, antibacterial applications, tissue engineering, etc. [5,11,23,24]. For the benefit of successful biomedical applications of GOs, an understanding of GO–kidney interactions is crucially required. In this study, we investigated the in vivo and in vitro interactions of PEGylated GOs with renal cells and the influence of the lateral size (~70 nm for *s*-GOs and ~300 nm for *l*-GOs) of GOs on them.

Using in vivo fluorescence imaging and in situ Raman imaging and spectroscopic analysis, we revealed that both *s*-GOs and *l*-GOs could be gradually eliminated from the kidney after i.v. injection; however, a fraction of injected GOs were deposited in the renal cortex at day 7 post-injection, suggesting glomerular or tubular accumulation of GOs. Particularly, *l*-GOs were frequently observed to deposit in the renal tubule by TEM imaging. Comparatively, a larger amount of *l*-GOs was deposited in the kidneys than *s*-GOs. Noteworthily, only the high dose of GO administration (15 mg/kg bw vs. 1 or 5 mg/kg bw) induced obvious histological and ultrastructural changes, especially in the glomeruli and renal tubules. The in vivo research suggests that the glomerulus and renal tubule are sensitive interaction target sites of GOs when they are eliminated from the kidney.

Therefore, we selected three typical human renal cells: human renal glomerular endothelial cells (HRGECs), human podocytes, and human renal proximal tubule epithelial cells (HK-2), which represent the major target cells that interact with exogenous substances during the renal clearance processes of filtration, secretion, and reabsorption. Importantly, these renal cells commonly involve in drug-induced kidney diseases including acute kidney injury (AKI), chronic kidney disease, etc. [14]. We demonstrated that the GOs induced differential toxicity to the three types of renal cells and that the lateral size of GO played a critical role when interacting with the cells. We found that when the renal cells were treated with 0.1~250 μg/mL *s*-GOs or *l*-GOs, high doses >50 μg/mL induced obvious time- and dose-dependent decreases in cell viability and increased cellular apoptosis accompanied by the elevation in intracellular LDH release and increase in oxidative stress, suggesting that the safe and biocompatible use of GOs should be at a relatively low dose.

Noteworthily, in this study, the *l*-GOs generally showed more pronounced cytotoxicity and more severe injury to the renal cells than *s*-GOs. The result is consistent with the previous findings, which suggest that the lateral size is one of the most important properties of GOs that greatly influences their in vivo and in vitro behaviors. Li and colleagues [25] found that GOs could induce lateral-size-dependent cytotoxicity in the Kupffer cells (KCs), i.e., the large-sized GOs led to more severe lipid peroxidation, aspase-1 activation, and GSDMD-mediated pyroptosis than the small-sized GOs. Liu et al. [29] demonstrated that the larger GOs triggered more robust interactions with toll-like receptors and more efficient NF-κB pathway activation in cells than the smaller GOs; in contrast, smaller GOs promoted greater M1 polarization, which was associated with inflammatory cytokine production and immune cell recruitment. The cytotoxicity of GOs is suggested by the interaction of GOs with the cellular membrane because previous studies have demonstrated that the larger-sized GOs could cause greater perturbation of the cellular phospholipid bilayer than the smaller GOs when GOs enter into the interior of the cells, such that larger lateral-sized GOs are more readily and rapidly internalized by the cell and lead to greater cytotoxicity [20,30,31].

Some previous studies have shown a trend of cell-type-related toxicity induced by nanomaterials [25,32]. Li et al. [25] found that GOs induced significantly more severe cytotoxicity in KCs than in the other two types of liver cells: the liver sinusoidal endothelial cells (LSECs) and hepatocytes, which are independent of the lateral size of GOs. Gies et al. [32] showed that GO was the least toxic to the adherent cell lines (e.g., NIH 3T3, U87, and A549) and the most toxic to the suspension cell lines (e.g., white blood cells NB4 and HL-60). However, more importantly, we did not find specifical renal cell-type-dependent cytotoxicity induced by GOs even though the HK-2 cells displayed the lowest cell viability and the highest intracellular LDH release with GO treatments of a high dose of 100 μg/mL because meanwhile, sensitive oxidative stress responses were also found to be induced in the HRGECs and podocytes. We believe the results will be helpful for a deeper understanding of GO interactions with renal cells when the 2D nanosheets are excreted in urine from the kidneys. Glomerular endothelial cells (GECs) and podocytes are important components of the glomerular filtration barrier (GFB), where GECs are a specialized microvascular cell type forming the inner part of the GFB and podocytes comprise filtration slits between foot processes. Therefore, drug-induced glomerular lesions generally involve endothelial permeability damage due to GEC injury and podocyte loss followed by the induction of chronic kidney diseases [33]. As for the proximal tubule epithelial cells (PTECs), they are most likely to be exposed to drugs in the processes of tubular excretion, concentration, and reabsorption after the drugs filtrate through the glomerulus; thus, PTECs are influenced greatly by drug toxicity. In this study, our results demonstrated that the GO-induced kidney injury was correlated with the interactions of GOs with the renal cells that are involved in the processes of renal clearance and furthermore suggested that the excretion obstacle of GOs could induce renal deposition of nanomaterials and subsequently cause nephrotoxicity.

Further investigation of the toxicological mechanism in HK-2 cells demonstrated that GO-induced cytotoxicity was associated with oxidative stress and an inflammation reaction. The results are similar to those of a previous study in which GOs induced dose-dependent cytotoxicity, including elevation in the oxidative stress level and a decrease in mitochondrial membrane potential in dental pulp stem cells [23]. Similarly, we revealed that a relatively high dose of GO treatments (50 or 100 μg/mL) induced oxidative stress in the HK-2 cells, which produced an increased level of ROS and decreased the antioxidative defense system due to the decrease in the GSH/GSSG ratio; although the Keap1/Nrf2 antioxidant system seemed to be activated, the elevated NOQ1 was not enough to scavenge the ROS. The elevated ROS was accompanied by inflammatory reactions to increase the levels of pro-inflammatory cytokines (e.g., TNF-α and IL-18), and mitochondrial dysfunction and final acceleration of cellular apoptosis. Moreover, the antioxidant function of Nrf2 could be inhibited by apoptotic engagement after DNA damage in cells [34,35]. Thus, in the presence of extensive cellular damage caused by high-dose GO-induced oxidative stress and cellular apoptosis, the downregulation of Nrf2 is hypothesized to involve in the molecular cross-talk between the Nrf2/Keap1 and apoptosis signaling pathways [33,35].

## 4. Methods

### 4.1. Preparation and Physiochemical Characterization of s-GOs and l-GOs

The small (*s*-GOs) and large (*l*-GOs) lateral-sized GOs were obtained using ultrasound-assisted exfoliation. The PEGylated GOs were synthesized according to our previous method [4]. Briefly, *s*-GOs and *l*-GOs were sonicated for 10 h and 10 min in an ice-water bath, respectively. The polyethylene glycol-modified GOs were prepared via EDC/NHS chemistry at room temperature. Then, the PEGylated *s*-GOs and *l*-GOs were purified by centrifugation, followed by dialysis for 12 h to remove free impurities.

Atomic force microscopy (AFM, AFM5500, Bruker, Billerica, UK) was used to characterize the thickness, size, and morphology of GOs. The Raman spectra of GOs were measured using a WITec Alpha 300 R Raman spectrometer (WITec, Ulm, Germany) with a 532 nm laser. The hydrodynamic diameters and zeta potential of GOs in deionized water or cell culture medium (DMEM) were measured using Zetasizer Nano ZS90 (MALVERN, Malvern, UK).

### 4.2. Animals and GO Treatments

Seven-week-old male C57bl/6N mice (~20 g body weight) were purchased from Beijing Vital River Laboratory Animal Technology Co., Ltd. (Beijing, China). All animal experiments were approved by the Institutional Animal Care and Use Committee. All the mice were housed in an air-conditioned room at 22 ± 2 °C and 45 ± 5% humidity, with a 12 h light–dark cycle with free access to food and deionized water. After a 7-day adaptation period, *s*-GOs and *l*-GOs were injected into the mice via tail vein at doses of 1, 5, and 15 mg/kg bw, which are comparable to the amounts commonly used in biomedical applications [36,37,38,39,40]. The control group mice were injected with a saline solution.

### 4.3. In Vivo Fluorescence Imaging of GO Distribution

Fluorescence imaging was performed using indocyanine green (ICG) as the fluorescent probe for the study of GOs’ in vivo distribution profile. Briefly, 0.1 mg/mL of ICG-NHS was mixed with 0.5 mg/mL GOs in DMSO solvent and then stirred at 4 °C in the dark for 3 h. Afterward, the mice were injected intravenously (i.v.) with *s*-GO/ICG or *l*-GO/ICG at a dose of 1 mg/kg bw. At 1, 4, 24, 48 h, and day 7 post-injection, the mice were sacrificed, and the organic tissues were collected. The tissues were visualized by 745 nm excitation and 820 nm emission using an IVIS Spectrum System (Perkin Elmer, Waltham, MA, USA).

### 4.4. In Situ Analysis of GO Renal Deposition by Confocal Raman Microscopy

GO deposition in the renal cortex was imaged by a WITec alpha 300 R confocal Raman microscope (WITec, Germany) with a 532 nm laser excitation source. Raman signals were acquired by a Peltier-cooled CCD (−70 °C) detector using a 600 line/mm grating spectrometer (UHTS 300, WITec, Ulm, Germany). Raman images were obtained at 0.5 × 0.5 µm^2^ pixel resolution at 500 ms/point integration time.

### 4.5. Fourier Transform Infrared Spectroscopy Analysis

Fourier transform infrared (FTIR) spectroscopy of the materials was performed on an iN10-IZ10 spectrophotometer (Thermo Fisher Scientific, Waltham, MA, USA) by using the KBr pellet method.

### 4.6. Transmission Electron Microscopy (TEM) Imaging

Fresh mouse kidneys were fixed overnight in 2.5% glutaraldehyde solution at 4 °C and then in a 1% osmium tetroxide solution for 3 h at 4 °C. After dehydration and resin embedding, the kidney specimens were cut to 70 nm thick slices and stained with uranyl acetate and lead citrate. Finally, the kidney samples were observed by TEM (JEOL JEM-1400).

### 4.7. Histological Analysis

Fresh mouse kidneys were fixed in 4% paraformaldehyde at room temperature for 24 h and then embedded in paraffin wax and sliced to a thickness of 6 μm, followed by hematoxylin–eosin (H&E) staining and Masson’s trichrome staining.

### 4.8. Cell Culture

The human renal glomerular endothelial cells (HRGECs), human podocyte primary cells, and human renal proximal tubule epithelial cells (HK-2) were obtained from Suzhou Bena Chuanglian Biotechnology Co. Ltd. HRGECs were cultured in high-glucose Dulbecco’s modified Eagle medium (DMEM; Biological Industries, Kibbutz Beit Haemek, Israel). Human podocytes were cultured in McCoy’s 5A modified medium (Biological Industries). HK-2 cells were cultured in Roswell Park Memorial Institute 1640 medium (RMPI 1640; Biological Industries). All culture media were added with 100 U/mL–100 μg/mL of penicillin–streptomycin (Gibco) and 10% fetal bovine serum (FBS; Biological Industries). Cells were incubated in a 95% humidity and 5% CO_2_ atmosphere at 37 °C.

### 4.9. Detection of Cell Viability

Cell viability was measured using the cell counting kit-8 (CCK-8) assay (Beyotime Biotech., Shanghai, China). Cells were seeded into 96-well plates at a density of 1.0 × 10^4^ cells per well, with five duplicate wells in each group. When 80–90% confluence was reached, cells were separately treated with *s*-GOs and *l*-GOs at 0.1, 1, 10, 50, 100, and 250 μg/mL for 6 h and 24 h. The dose of 0.1~250 μg/mL was selected based on previous studies that reported that the IC50 value of GO cytotoxicity is approximately within the range from 50 to 100 μg/mL [41,42]. The relative cell viability was calculated as the percentage of untreated cells.

### 4.10. Analysis of Cell Apoptosis

The cell apoptosis analysis was performed by flow cytometry using the Annexin V (AV)-FITC/PI double staining method. Cells were seeded in 6-well plates and incubated with GOs at 10, 50, and 100 μg/mL for 24 h. After treatment, cells were collected and washed by PBS three times, followed by incubation with AV-FITC and PI solution (Beyotime Biotech., Shanghai, China) and detection by flow cytometry. The cells undergoing apoptosis were represented by the ratio of AV-positive cells and PI-positive cells.

### 4.11. Detection of LDH Release in Treated Renal Cells

Cells were incubated into 96-well plates at a density of 1.0 × 10^4^ cells per well overnight. Then, the culture medium was replaced by a fresh one containing with *s*-GOs and *l*-GOs at concentrations of 0, 0.1, 1, 10, 50, and 100 μg/mL. After 6 h and 24 h of incubation, the 96-well plates were centrifuged, and the supernatants were collected. The amounts of LDH release were measured by an LDH cytotoxicity assay kit according to the manufacturer’s instructions.

### 4.12. Measurement of Intracellular ROS, GSH, and GSSG

Cells were seeded into 6-well plates at 3 × 10^5^ cells per well and incubated for 24 h to allow the attachment of cells. Then, the cells were pretreated with 1, 10, 50, and 100 μg/mL of *s*-GOs and *l*-GOs for 2 h. For reactive oxygen species (ROS) measurement, DCFH-DA (10 µM) was added with further incubation in darkness for 30 min.

The levels of total glutathione (T-GSH), reduced GSH, and oxidized GSG (GSSG) were assessed with a glutathione reductase-DTNB (5,5′-dithobis-2-nitrobenzoic acid) recycling assay using a GSH/GSSG quantification kit (Beyotime Biotech., China). Cells were all incubated in 6-well plates at a density of 4 × 10^5^ cells per well. The absorbance of the GSH chromogenic product was read at 412 nm. Each sample was analyzed in three pairs of wells.

### 4.13. Measurement of Cellular Uptake of GOs

HK-2 cells were separately co-cultured with 10 μg/mL of FITC-labeled *s*-GOs and *l*-GOs for 4 and 24 h. Cellular uptake of *s*-GO/FITC and *l*-GO/FITC was observed under confocal fluorescence microscopy (Nikon, LU-M4, Tokyo Metropolis, Japan). The nuclei of the cells were stained with DAPI (4′,6-diamidino-2-phenylindole).

### 4.14. Detection of Mitochondrial Membrane Potential

The mitochondrial membrane potential (Δψm) was detected by JC-1 dye (Beyotime Biotech., China). The fluorescence emission shift from red to green indicates mitochondrial depolarization. HK-2 cells were incubated in glass dishes for 12 h and then treated with 100 μg/mL of *s*-GOs and *l*-GOs for 4 h. Afterward, cells were washed once with PBS, and 1 mL of JC-1 staining working solution was added and incubated for 20 min at 37 °C. The cells were then observed under confocal fluorescence microscopy (Nikon, LU-M4).

### 4.15. RNA Extraction and Quantitative RT-PCR Analysis

Total RNA was extracted from the HK-2 cells using Trizol reagent (Beyotime Biotech., Shanghai, China). The extracted RNA was quantitatively determined using NanoDrop One Microvolume UV-vis Spectrophotometer (ThermoFisher, Waltham, MA, USA). The mRNA expressions for human *KEAP1*, *NRF2*, *NQO1*, *NOX2*, *TNFA, IL18, IL1B,* and *IL6* were analyzed using BeyoFast™ SYBR Green qPCR Mix (Beyotime Biotech., Shanghai, China) on a CFX Connect Real-time PCR system (Bio-RAD, Hercules, CA, USA). Primer sequences are shown in Supplementary Table S1. GAPDH was used as a reference gene. Relatively quantitative levels of samples were determined by the 2^−ΔΔCT^ method.

### 4.16. Statistical Analysis

All data are presented as mean ± standard deviation. Data were analyzed using Student’s *t*-test or one-way analysis of variance (ANOVA) to compare the statistical differences between groups. A value of *p* < 0.05 was considered statistically significant. Significant levels were set at *^, #^*p* < 0.05, **^, ##^*p* < 0.01, and ***^, ###^ *p* < 0.001.

## 5. Conclusions

In this study, we explored the lateral dimension-, dose- and time-dependent deposition and injury of PEGylated GOs in individual renal compartments in vivo after i.v. injection and the cytotoxic effects in three typical human renal cells: HRGECs, podocytes, and HK-2. We revealed that glomeruli and renal tubules are sensitive target sites when GOs are eliminated from the kidney, but only the high dose of GO administration induced obvious histological and ultrastructural changes in the kidneys. We showed that the *l*-GOs generally presented more severe cytotoxicity and more severe cellular injury than the *s*-GOs, demonstrating lateral size-dependent toxicity to the renal cells. We also demonstrated that GO-induced cytotoxicity involved oxidative stress and an inflammation reaction and mitochondrial damage-induced apoptosis. Importantly, no renal cell-type-dependent cytotoxicity was found by GO induction, suggesting that more attention should be paid to the ability of a high dose of GOs to cause renal deposition and the thereafter induced nephrotoxicity.

## Data Availability

The data that support the findings of this study are available from the corresponding author upon reasonable request.

## Supplementary Materials

The following are available online at https://www.mdpi.com/article/10.3390/molecules27227956/s1, Table S1-Sequences of primers for RT-qPCR analysis. Figure S1: TEM images show the *l*-GOs deposited in the renal interstitial capillaries after i.v. injection of 15 mg/kg bw *l*-GOs. Figure S2: Cell viability assays of HRGECs, podocytes, and HK-2 cells following 0.1, 1, 10, 50, 100, and 250 μg/mL doses of *s*-GO and *l*-GO treatments for 6 h. Figure S3: Annexin V-FITC/PI flow cytometry for the detection of cell apoptosis after cells were treated with 10 and 50 µg/mL *s*-GOs and *l*-GOs for 24 h. Figure S4: LDH leakage in HRGECs, podocytes, and HK-2 cells after cells were treated with *s*-GOs and *l*-GOs for 6 h.

## Sample Availability

Samples of the compounds are not available from the authors.
