# Supplementary material for: Understanding the Role of the Lateral Dimensional Property of Graphene Oxide on Its Interactions with Renal Cells
Source: Molecules. 2022 Nov 17;27(22):7956. doi: 10.3390/molecules27227956 (PMC9697150; doi:10.3390/molecules27227956)
Supplement: Supplementary file 1 [file molecules-27-07956-s001.zip › molecules-2015831-supplementary.pdf]

Supplementary Materials

Understanding the Role of the Lateral Dimensional Property of Graphene Oxide on its Interactions with Renal Cells

Wei Chen[1,2], Bing Wang[1,*], Shanshan Liang[1,2], Meng Wang[1], Lingna Zheng[1], Si Xu[1,3], Jiali Wang[1,3],Hao Fang[1,2], Pu Yang[1,2], Weiyue Feng[1,*]

[1] CAS Key Laboratory for Biomedical Effects of Nanomaterials and Nanosafety, Institute of High Energy Physics, Chinese Academy of Sciences, Beijing 100049, China

[2] University of Chinese Academy of Sciences, Beijing 100049, China

[3] School of Pharmacy in Yantai University, Yantai, 264005, China

* Correspondence, wangbing@ihep.ac.cn; fengwy@ihep.ac.cn
Tel.: +86-010-88233209

Table S1. Sequences of primers for RT-qPCR analysis

Genes	Forward primers (5'-3')	Reverse primers (5'-3')
KEAP1	CTGGAGGATCATACCAAGCAGG	GGATACCCTCAATGGACACCAC
NRF2	CAACTACTCCCAGGTTGCCC	TGGGGTTTTCCGATGACCAG
NQO1	GCTGGTTTGAGCGAGTGTTC	CTGCCTTCTTACTCCGGAAGG
NOX2	ACCTCAACTGCAGCCTTATC	ATCCAACAATCTCCTGGTTCTC
TNFA	AGCCCATGTTGTAGCAAACCC	GTAGGCCCCAGTGAGTTCTG
IL1B	GCCAATCTTCATTGCTCAAGTGT	AGCCATCATTTCACTGGCGA
IL6	CCTTCGGTCCAGTTGCCTTC	CACCAGGCAAGTCTCCTCATT
IL18	ATCGCTTCCTCTCGCAACAA	GTCCGGGGTGCATTATCTCT
GAPDH	ACCAAATCCGTTGACTCCGAC	CTCCTGTTCGACAGTCAGCC

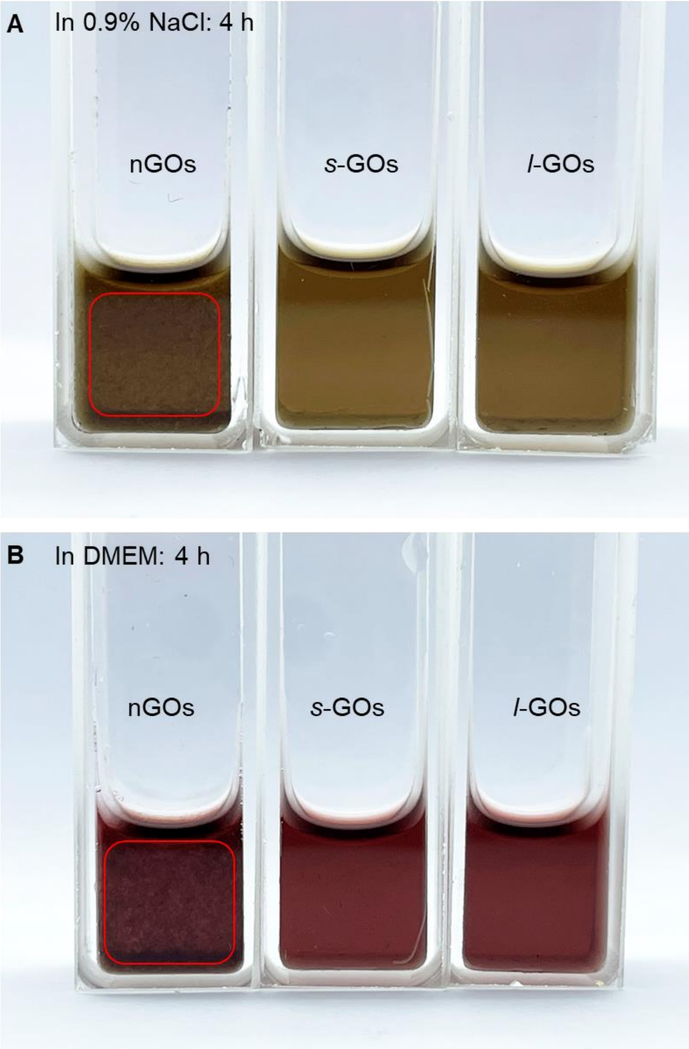

Figure S1. The photographs of nGOs, s-GOs and l-GOs dispersed in 0.9% NaCl (A) and DMEM (B) at 4 h. nGOs: non-pegylated GOs. Red square: GOs cluster formed by unstable.

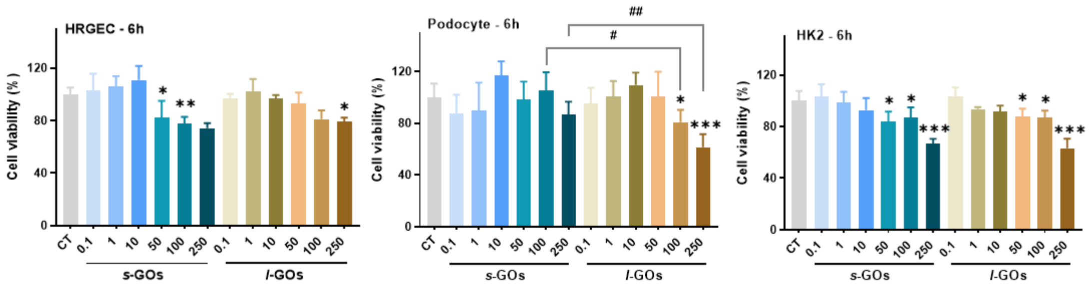

Figure S2. Cell viability assays of HRGEC, podocytes and HK-2 cells following 0.1, 1, 10, 50, 100, and 250 µg/mL doses of *s*-GO and *l*-GO treatments for 6 h.

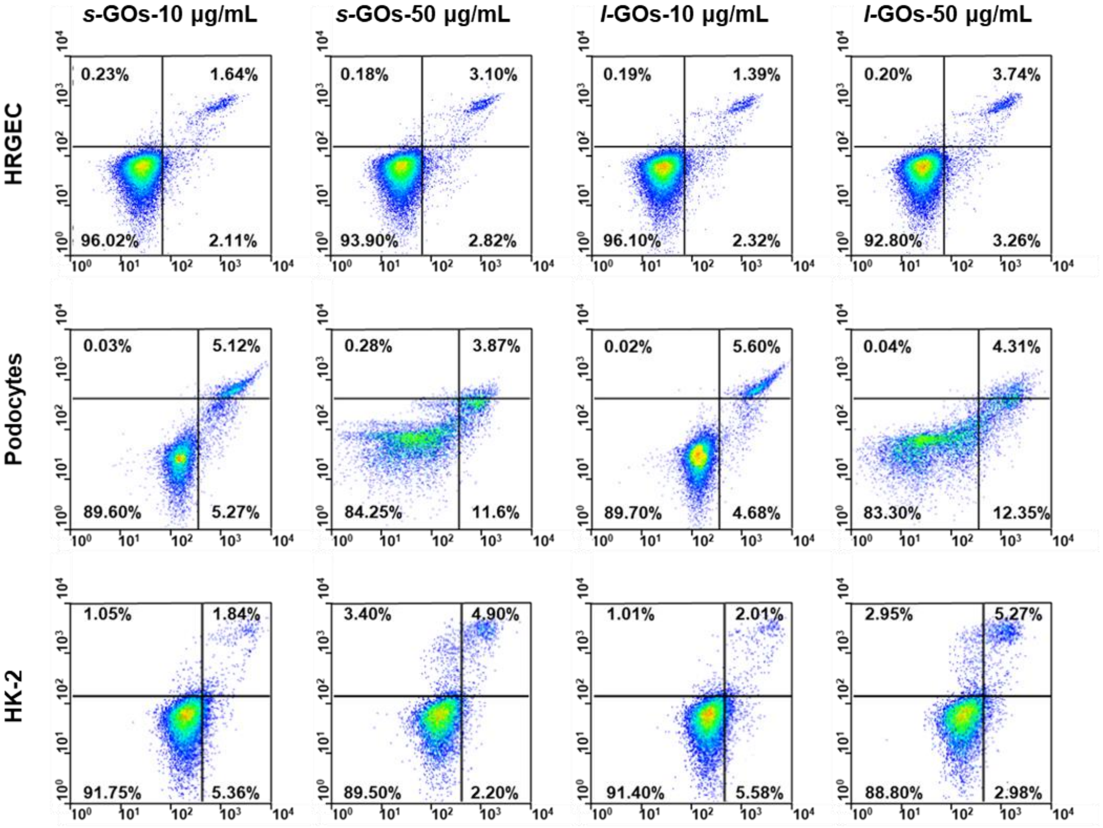

Figure S3. Annexin V-FITC/PI flow cytometry for the detection of cell apoptosis after cells were treated with 10 and 50 μg/mL *s*-GOs and *l*-GOs for 24 h.

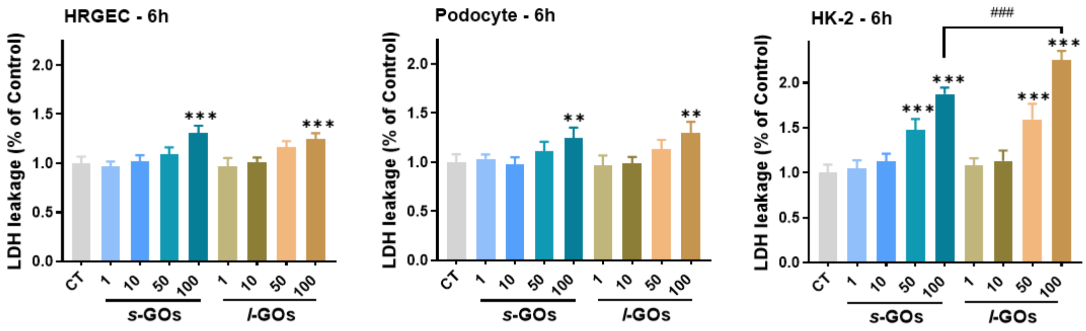

Figure S4. LDH leakage in HRGEC, podocytes and HK-2 cells after cells were treated with *s*-GOs and *l*-GOs for 6 h.
